# The impact of social phobia on willingness to communicate in a second language: The mediation effect of ideal L2 self

**DOI:** 10.3389/fpsyg.2022.1018633

**Published:** 2022-11-03

**Authors:** Chen Zhang, Wenzhong Zhang

**Affiliations:** College of Foreign Languages, Nankai University, Tianjin, China

**Keywords:** social phobia, L2 willingness to communicate, ideal L2 self, mediation effect, Psychopathology

## Abstract

In recent years, a greater focus has been placed on the influential power of domain-general psychological properties in second language acquisition and learning. The investigations of these properties, such as grit, academic procrastination and enjoyment etc. have been extensively conducted and are well-documented. Notwithstanding the surge of academic inquiry, the link between psychopathological notions and second language learning has not been adequately established and thoroughly scrutinized. The current study, therefore, aims to broaden the spectrum of second language research and explore the impact of social phobia on willingness to communicate in the second language context. Meanwhile, this research introduces the self-construct, particularly the ideal L2 self to further examine and elucidate the impact. 173 qualified Chinese speakers of L2 English participated in the study. By conducting correlation analysis, regression analysis and structural equation modelling analysis, it was revealed that social phobia had a significant negative impact on L2 willingness to communicate in social situations of meetings and public speaking. Ideal L2 self acted as a complete mediating role in the impact. Pedagogical implications and future directions were proposed and discussed.

## Introduction

Over the past decades, studies on individual differences in second language acquisition research have experienced unprecedented growth (Saito, 2018; Wallace, 2022). Research concerning affective and psychological differences has demonstrated rapid and remarkable advances. Properties such as L2 anxiety, L2 motivation and L2 self-efficacy etc. have been the subjects of much systematic investigation and are instrumental in our understanding of how second language abilities are acquired and developed (Papi et al., 2019; Oh, 2022; Sparks and Alamer, 2022). Although research on these domain-specific psychological properties is conducive and constructive, without an overlook from a holistic perspective, relevant examinations could only provide a snapshot of the influential mechanism of individual psychological differences. Scholarly attention has thus been steered to a much broader exploration of domain-general psychological properties in the L2 context more recently. Grit (Liu and Wang, 2021), resilience (Li, 2022), academic procrastination (Zhang and Zhang, 2022) and enjoyment (Dewaele and Alfawzan, 2018) etc. were at the centre of the discussion. However, domain-general psychopathological properties were still empirically understudied in second language learning. Typically, social phobia, an important psychopathological property, has rarely been studied in the L2 context. Social phobia is deeply rooted in communication in social situations, and so is second language learning (Amsbary and McCroskey, 1967; Junn, 2021). However, up to now, the mechanism of the association between the two has not been thoroughly probed.

Furthermore, it is worth mentioning that social phobia is featured by behavioural inhibition and social withdrawal (Neal et al., 2002). In contrast, L2 willingness to communicate (WTC) and ideal L2 self are strongly characterized by behavioural intention and are the most motivational psychological properties in the L2 context. However, the question of whether, how and in what way social phobia would undermine these two L2 psychological properties remained unanswered. The vital influential path has also not been detected and affirmed yet.

Hence, the current study introduces psychopathological properties to the second language learning context and exhibits to be interdisciplinary. By examining the mediating role of the ideal L2 self construct, this research aims to further understand the impact of social phobia on L2 WTC and shed light on the influential trajectory. The results of this work would generate robust empirical evidence and provide new insights for research and pedagogy.

## Literature review

### Social phobia

Social phobia, also called social anxiety disorder, is a chronic psychopathological condition that denotes great fear in social situations (World Health Organization, 2019). It is in fact omnipresent for people to feel apprehensive and alarmed in social settings. However, social phobia is beyond the boundary of normality. It is featured by marked, intense and excessive fear, which might lead to dysfunction and distress in daily activities, according to the 11th version of the International Classification of Diseases (ICD-11; World Health Organization, 2019). People with social phobia usually show considerable concerns regarding potential negative evaluations from others. They may also feel deeply embarrassed and humiliated when their actions are observed and scrutinized in social interactions and performances. Many sufferers display protracted clinical symptoms, encompassing blushing, sweating, trembling, palpitation, and even panic attacks (Beidel and Randall, 1994). Social phobics usually have quite an early onset of this mental condition which could occur in childhood and teenage time. Most of them endure the symptoms during their personal growth, which would significantly impede their development in social life (World Health Organization, 2019). Social phobia differs from communicative anxiety in the foreign language setting since social phobia is a domain-general psychopathological phenomenon that can be seen in various scenarios and contexts. However, communicative anxiety in a foreign language setting is typically domain-specific and only appears when people communicate in a foreign language.

After illustrating the definition of social phobia, social phobia’s features and current situations worldwide, its aetiology, its theoretical foundation regarding the self-construct and its influences in academic performances and relevant L2 contexts are reviewed in the following paragraphs.

Substantial fear would lead to behavioural inhibition and social withdrawal in social phobia (Neal et al., 2002). Most recently, since the Covid-19 pandemic has stimulated a remarkable development of online interactions, people who have a propensity for social phobia could find their sanctuary in online communications. This would seemingly mitigate their symptoms in a short term but would undoubtedly aggravate the situation where they are required to communicate face-to-face or perform offline in the long run (Morrissette, 2021). In view of the existing literature that social phobia is now perplexing an increasing number of people worldwide (Ohayon and Schatzberg, 2010), it is a psychopathological issue that needs to be adequately addressed.

Etiologically, social phobia may arise from a combination of complex biological, environmental and cultural factors. Apart from possible inherited traits, temperament and brain structures, the major cause also includes a prior traumatic social experience or unfavourable parenting styles (Spence and Rapee, 2016). Though sharing similarities in symptomatology and behavioural inhibition (Kagan, 1999), social phobia is discrepant from the concept of shyness, in that shyness is much related to a transitory condition in a specific context where the intensity of fear and social withdrawal are not underlined, and it would not provoke impaired functioning (Heiser et al., 2003). Social phobia is clinically correlated with general anxiety, obsessive–compulsive disorder, avoidant personality disorder and depression etc. and could be comorbid with these psychological disturbances. Psychotherapy, such as cognitive behavioural therapy and medication, are the two mainstream protocols to treat social phobia.

In the theoretical elucidation of social phobia, several cognitive-behavioural models were established to further understand its gist and internal mechanism, among which the self-construct was specifically highlighted. The self is regarded as a systemized repository, or cognitive schema, which contains the beliefs, thoughts, prior experiences etc. of oneself, and could generalize and help navigate information processing and practice construal (Ellemers et al., 2002). The cognitive model raised by Clark and Wells (1995) and later improved by Clark (2001) postulated that social phobics strive to fulfil the expectations of others but, unfortunately, fall short of the capacity to achieve. People with social phobia hold a series of assumptions before entering social settings, such as the “excessively high standards for social performances,” “conditional beliefs concerning the consequences of performing in a certain way” and “unconditional negative beliefs about the self” (Clark, 2001: 406). These assumptions would trigger negative self-processing when regarding the self as a social object, thus gloomy self-images would be further generated. Safety behaviour, as a type of maladaptive coping behaviours, is a critical element in illustrating social phobia in this model. It is an internal mental process that prompts self-focused attention and negative self-processing and finally forms a vicious circle. When people with social phobia step out of the feared situation, they usually ruminate about the social failure that they have experienced. Consequently, an unremitting negative self-appraisal is settled. Rapee and Heimberg (1997) model accentuated the mismatch between the distorted self-image and the perceived anticipation from others, which eventually led to internal anxious perception. Hofmann (2007) further acknowledged this argument and posited that social phobia arose when people had social phobia set extremely high standards but had difficulties in understanding whether the goals were attainable or not. When failure is predetermined, social phobics would overestimate the possible unsatisfactory outcome and lose control of the social encounter. Stopa (2009) proposed a tripartite approach to conceptualizing social phobia, due to the inadequacy and incomprehensiveness of the above models. Self-content (e.g., self-images, beliefs about the self, self-esteem), self-structure (e.g., self-organization, self-concept clarity) and self-process (e.g., self-awareness, self-evaluation, social/temporal comparison) are the crucial elements. Built on this approach, Alden et al. (2014) added the fourth component, self-related motives and behavioural strategies, to facilitate the comprehension of the mechanism of social phobia. Typically, social phobia is featured by distorted self-images (Rapee and Heimberg, 1997), maladaptive self-beliefs (Clark and Wells, 1995), lower self-esteem (Rasmussen and Pidgeon, 2010), lower self-concept clarity (Stopa et al., 2010), negative self-evaluation (George and Stopa, 2008), behavioural inhibition (Neal et al., 2002) and post-event rumination (Abbott and Rapee, 2004) etc.

Considering the nature of social phobia, researchers have identified its impact in extensive circumstances, among which the academic context received special attention. Predominantly, social phobia could substantially impair academic performances, on account of existing empirical evidence. Baptista et al. (2012) studied the prevalence of social phobia among Brazilian university women students and the academic impairment which might be induced correspondingly. It was manifested that the female social phobics who participated in the study demonstrated significantly lower academic performances than those without social phobia. In the same vein, Leigh et al. (2021) investigated the route from social anxiety to educational achievement via concentration. By analysing the results from a sample of 509 secondary school students in the United Kingdom, it was revealed that adolescents with social phobia generally had concentration problems, which might lead to unsatisfactory academic outcomes. Vilaplana-Pérez et al. (2021) observed a similar negative association between social phobia and educational performance. Researchers also detected that the adverse impact of social phobia would remain enduring across the lifespan. Archbell and Coplan (2022) also discovered that social phobia was negatively correlated with the academic communication with instructors.

Given that L2 learning is a critical part of the educational systems worldwide, it is highly possible that social phobia might influence L2 learning in a negative way. Since language learning is expressive, communicative, and deeply grounded in social contacts, L2 learners with social phobia would probably encounter formidable obstacles in L2 performances and interactions. King (2013a,b) explored the role of social anxiety in Japanese foreign language classrooms. A prominent relationship between social anxiety and the avoidance of talk among foreign language learners was detected. However, other than this piece of clue, little empirical evidence has been obtained to date and the experimental support is far from adequate, especially regarding the potential influence of social phobia on L2 interactions. Thus, how social phobia would influence L2 learning is an issue that needs rigorous examination and persistent investigation.

### Willingness to communicate in a second language

The concept of willingness to communicate was initially proposed in the first language context and was framed as “a personality-based, trait-like predisposition” (McCroskey and Baer, 1985: 6). MacIntyre et al. (1998) introduced this concept to the L2 context and contended that WTC is not limited to be trait-like but can be “situational,” and defined L2 WTC as “a readiness to enter into discourse at a particular time with a specific person or persons, using an L2” (MacIntyre et al., 1998: 547). They further devised a heuristic pyramid model to understand the factors that could dramatically impact L2 WTC. The potential determinants of L2 WTC were classified into six layers in the conceptualization. The first three layers comprised transient situation-specific factors and the latter three layers consisted of enduring influences. Communication behaviour (i.e., L2 use), behavioural intention (i.e., willingness to communicate) and situated antecedents (i.e., the desire to communicate with a specific person, state communicative self-confidence) were formulated in the first three layers. Meanwhile, motivational propensities (i.e., interpersonal motivation, intergroup motivation, self-confidence), affective-cognitive context (i.e., intergroup attitudes, social situation, communicative competence) and social and individual context (intergroup climate, personality) were considered as long-lasting factors and were constructed in the latter three layers, respectively. This theoretical model yielded a comprehensive illustration of L2 WTC and is the guidance for further explorations.

L2 WTC is in nature complex and dynamic (Pawlak and Mystkowska-Wiertelak, 2015; MacIntyre and Wang, 2021). Rather than being independent, it is closely associated with psychology, educational backgrounds, and communicative competence, etc. L2 learners’ level of WTC might fluctuate during the learning span and can be hugely influenced by numerous factors.

Since the work of MacIntyre et al. (1998), the study of L2 WTC and its conceivable determinants has gained momentum. Much empirical evidence based on quantitative and qualitative data has been accumulated to demonstrate the power of the proximal antecedents on L2 WTC. As the representatives of the quantitative studies, Peng and Woodrow (2010) highlighted the roles of classroom environment, motivation and communication confidence, etc., in promoting L2 WTC and observed the direct and indirect effect of motivation. Piechurska-Kuciel (2018) has probed into the predicting effect of openness to experience on L2 WTC and found it to be immediately relevant and directly significant. Shirvan et al. (2019) conducted a meta-analysis in terms of the key variables which might influence L2 WTC. By including a sample of 22 studies, the researchers found that perceived communicative competence, language anxiety and motivation correlate significantly with L2 WTC. Ito (2021) focused on illustrating the psychological network in L2 WTC and reiterated the strong link between perceived communicative competence and L2 WTC. Dewaele and Pavelescu (2021) pinpointed the dynamic nature of the correlation between foreign language enjoyment and foreign language anxiety and acknowledged the influences of learners’ personalities and experiences on shaping L2 emotions. Meanwhile, the qualitative research administered by Peng (2012) revealed six immediate antecedents in the microsystem of L2 WTC in classrooms, involving learner beliefs, motivation, cognitive factors, linguistic factors, affective factors and classroom environment.

Overall, these studies provided important insights into the predictors of L2 WTC, particularly in the social, psychological, and personality-related arena. However, remarkably little research has examined the impact of potential psychopathological properties on L2 WTC. It is therefore particularly urgent to elicit relevant robust and corroborative evidence and contribute to this interdisciplinary investigation.

### Associating social phobia and L2 WTC: Ideal L2 self as a bridge

Investigating the impact of social phobia on L2 WTC is critical, since behavioural withdrawal, which is the most prominent feature of social phobia, could possibly undermine L2 WTC (MacIntyre et al., 1998).

When exploring how social phobia would be associated with L2 WTC, ideal L2 self is presumably an overpass which could link these two concepts. It is one of the most motivational properties in L2 learning as well (Dörnyei and Ushioda, 2011) and can be well compared with the qualities of the self-construct regarding social phobia. Ideal L2 self is the reification of the ideal self in a second language context and is an integral part of the L2 motivational self system. With regard to ideal self, it is a concept that originated from the theory of possible selves (Markus and Nurius, 1986) and self-discrepancy theory (Higgins, 1987, 1998). Markus and Nurius (1986) posited that possible selves were rooted in the self from the past and involved the portrayal of future selves. Possible selves embodied individuals’ perceptions regarding “what they might become, what they would like to become and what they are afraid of becoming” (Markus and Nurius, 1986: 954). Various types of possible selves, including positive selves (e.g., hopeful self) and negative selves (e.g., dreaded self), were envisioned and proposed. These possible selves are crucial in that they are the impetus for shaping relevant approaching or avoiding behaviours in the future. Thus, possible selves could act as a future self guide to direct prospective reactions and witness self-changing dynamics. Built on the theory of possible selves, Higgins (1987, 1998) further specified the notion of possible selves and proposed that ideal self, which denotes the attributes that individuals would ideally like to possess, is one of the critical components according to the self-discrepancy theory (Higgins, 1987: 320).

Several lines of evidence suggest that ideal self is aspirational (Boyatzis and Dhar, 2022) and is a prominent driver for intentional change (Boyatzis and Akrivou, 2006). Ideal self has the nature of being promotive, which indicates the acquiring propensity of hopes, advancements etc., rather than being preventive or avoiding possible negative outcomes (Higgins, 1998). Motivation could then be initiated by minimizing the discrepancy between one’s actual self and ideal self. Thus, it can be inferred that ideal self is closely associated with behavioural intention, instead of behavioural inhibition. It is also worth noting that this motivational capacity of ideal self is grounded in the conditions of a desired, plausible, challenging but attainable, elaborate and vivid self-images (Dörnyei and Ushioda, 2011). Hence, it is tempting to speculate that ideal self is closely related to positive and clear self-images.

As illustrated previously, social phobia is strongly featured by behavioural inhibition and social withdrawal (Neal et al., 2002). Most importantly, social phobic usually had distorted self-images (Rapee and Heimberg, 1997) and negative self-evaluation (George and Stopa, 2008). This is in stark contrast with the qualities demonstrated by ideal self, whose core traits are intentional, promotive, motivational. In light of this, it is highly possible that social phobia is negatively correlated with ideal self. Since ideal L2 self is the reified form of ideal self in the L2 context, a negative relation between social phobia and ideal L2 self could be foreseen. Empirical evidence is awaiting to be collected to demonstrate this assumption.

Recent studies have also informed our knowledge of the relationship between ideal L2 self and L2 WTC. Munezane (2013) pioneered in investigating the direct link between the two variables and found that ideal L2 self could significantly and positively predict L2 WTC. The following works conducted by Öz (2016), Lee and Lee (2020), Shen et al. (2020), Lan et al. (2021) and Zhang et al. (2022) reiterated this prominent connection.

On account of the bridging power of ideal L2 self, the influential capacity of social phobia on L2 WTC could be conceivably explored and the corresponding pathways could be properly examined. Therefore, the current study seeks to address the potential impact of social phobia on L2 WTC and assess the extent to which ideal L2 self contributes to this impact.

## Research questions

The current research aimed to investigate the influential power of social phobia on L2 WTC and test the mediation effect of ideal L2 self in the impact.

Specific research questions were:

1. How would social phobia influence L2 WTC?
2. Can ideal L2 self act as a mediating role in the impact of social phobia on L2 WTC?

## Methodology

### Participants

Initially, through convenience sampling and snowball sampling, 184 Chinese speakers of L2 English were invited to participate in this research. The participants were undergraduate students from a renowned national key university in north China. A vast majority of the participants had the background of language studies, which involved English, Japanese, French, Russian, German, Spanish, Portuguese, Italian and Arabic. The researchers first invited students who enrolled in the courses they offered and asked these participants to identify further appropriate members who could and wished to participate in the research. Two criteria were set to assess the validity of the data from the participants. Firstly, similar questions were involved in the set of questionnaires, but the questions were raised in a reversed manner. If participants consistently provided contradictory answers in similar questions throughout the response, their data were regarded invalid. Secondly, if participants consistently chose the extreme values (i.e., the highest value or the lowest value) according to each subscale, their answers were treated as invalid. After rigorously checking the answers and feedback in the questionnaire survey, data from 173 participants (Male = 29, Female = 144) remained whereas the data from the other 11 participants were eliminated due to invalidity. The mean age of the remained participants was 18.1 (*SD* = 0.66).

### Instruments

The current study utilized a set of three questionnaires, encompassing Liebowitz social anxiety scale (LSAS), the willingness to communicate scale and a questionnaire on L2 motivational self system, to fully explore the research questions. They were conducted to measure the level of social phobia, L2 WTC and ideal L2 self, respectively.

#### Liebowitz social anxiety scale

Liebowitz social anxiety scale (LSAS) is a classical and well-received questionnaire in psychopathology, developed by Liebowitz (1987) and was designed to assess the levels of social phobia. 24 items were included in LSAS and can be repetitively adopted in the examination of social phobia in terms of fear and avoidance, respectively. Among the 24 items, 13 items were devised to explore social phobia in performances and the other 11 items were constructed to investigate social phobia in social interactions. A 4-point Likert scale (Fear subscale: 0 = none, 1 = mild, 2 = moderate, 3 = severe; Avoidance subscale: 0 = never, 1 = occasionally, 2 = often, 3 = usually) was adopted to rate the items in the questionnaire. The overall score of LSAS could fall within the range of 0–144. Mennin et al. (2002) and Rytwinski et al. (2009) proposed that an overall score of 30 could be a cut-off point to differentiate people with social phobia from healthy controls. An overall score between 30 and 60 indicates non-generalized social phobia (i.e., individuals feel anxious in a limited range of social situations), and the score range between 60 and 90 suggests generalized social phobia (i.e., individuals feel anxious in most social situations). Scores higher than 90 imply that social phobia is highly possible.

Due to its high reliability and validity demonstrated, LSAS has been widely utilized in empirical research and clinical diagnosis. In the current study, the highly reliable (Fear subscale: Cronbach’s α = 0.958; Avoidance subscale: Cronbach’s α = 0.967) and valid features of construct (Fear subscale: KMO = 0.932, *p* < 0.001; Avoidance subscale: KMO = 0.943, *p* < 0.001) have been further verified.

#### The willingness to communicate scale

The willingness to communicate (WTC) scale was established to evaluate the level of WTC in different social situations (McCroskey, 1992). Instead of tackling associated feelings of apprehension and introversion from an indirect perspective, the WTC scale directly undertook inspections in the approaching and avoiding propensity in communication. It comprises 20 items in total, within which 8 items are fillers. The 12 test items can be utilized to demonstrate the level of WTC concerning three types of audiences (i.e., stranger, acquaintance and friend) and four types of communication contexts [i.e., interpersonal conversations, (small) group discussion, (large) meetings and public speaking]. Given complete free choice, the survey takers could rate on a continuum of 0 (never)–100 (always). According to Shaffer (2021), an overall score above 82 represents a high level of WTC. The score range between 52 and 82 indicates a medium level. An overall score below 52 implies a low level of WTC.

Adequate reliability and validity of the scale were exhibited in the current study (Cronbach’s α = 0.951; KMO = 0.928, *p* < 0.001). Since the WTC scale was not specifically designed for the second language context, when administering this scale in the current study, the participants were informed to rate their WTC by using their L2 English. The WTC scale has been frequently adopted in second language research (e.g., Cao and Philp, 2006; Alemi et al., 2013), thus it is feasible to be administered in the current study as well. Considering that social phobia may arise in different social situations, the measurement of the WTC scale was based on the classification of communication contexts in this research.

#### Questionnaire on L2 motivational self system

The questionnaire of assessing the level of ideal L2 self in the current study was extracted from the survey conducted in Papi (2010), which was further developed from Taguchi et al. (2009). Originally, in Papi’s questionnaire (2010), 5 sections, including ideal L2 self, ought-to L2 self, English learning experience, intended effort and English anxiety, were designed. Given the specific aim of exploration in the current study, only the ideal L2 self section was retained. 6 items were involved in this section. A 6-point Likert scale (from 1 “strongly disagree” to 6 “strongly agree”) was taken as the measurement. As defined by Oz (2015), the rated score above 4.25 indicates a high level of ideal L2 self. The score range between 2.5 and 4.25 represents a moderate level and the range between 1 and 2.5 suggests a low level of ideal L2 self. This section of the questionnaire was reported to have a sound reliability (Cronbach’s α = 0.77) in Papi (2010).

### Research procedures

The recruited participants were informed of the task requirements at the beginning. They were then invited to rate the levels of their social phobia, L2 WTC and ideal L2 self in a series of questionnaires in one session. The generated data were incorporated in further statistical analysis.

## Data analysis and results

### Descriptive statistics

**Figure 1:**
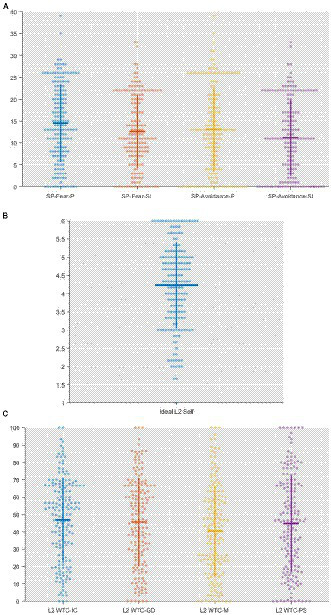
**(A)** Beeswarm plot – Social phobia. SP: Social phobia; P: Performance; SI: Social interactions. **(B)** Beeswarm plot – Ideal L2 self. **(C)** Beeswarm plot – L2 WTC. IC: Interpersonal conversations; GD: Group discussion; M: Meetings; PS: Public speaking.

Descriptive analyses of the questionnaire data were processed by *SPSS 26*, to construct a comprehensive picture of the received answers. Produced by *Matlab*, Figures 1A–C are the Beeswarm plots which show the data distribution.

In terms of the examination of social phobia, participants demonstrated non-generalized social phobia (*Mean of the overall score* = 51.41, *SD of the overall score* = 32.95). The score distributions in both the sub-scales of fear and avoidance, concerning performances (Fear subscale: *Mean* = 14.51, *SD* = 8.85; Avoidance subscale: *Mean* = 13.09, *SD* = 9.61) and social interactions (Fear subscale: *Mean* = 12.6, *SD =* 8.35; Avoidance subscale: *Mean* = 11.2, *SD* = 8.72) are presented in Figure 1A. Additionally, it was manifested in Figure 1B that participants had a moderate level of ideal L2 self (*Mean* = 4.23; *SD* = 1.179). Furthermore, participants demonstrated a low level of L2 WTC (*Mean of the overall score* = 44.47 < 52, *SD of the overall score* = 22.84). The data distributions in the contexts of interpersonal conversations (*Mean* = 46.97; *SD* = 23.84), group discussion (*Mean* = 45.65; *SD* = 25.53), meeting (*Mean* = 40.57; *SD* = 26.08) and public speaking (*Mean* = 44.70; *SD* = 27.78) of L2 WTC are shown in Figure 1C. The obtained data of each variable conformed to the normal distribution (Kline, 2016).

### Correlation and regression analyses

Based on the understanding of the data distribution, correlation analyses and a series of regression analyses were conducted, to provide information in terms of the inter-correlations and casual relationships among the variables of social phobia, ideal L2 self and L2 WTC.

As displayed in Table 1, all the four sub-scales of social phobia were negatively correlated with ideal L2 self. It was also denoted that the sub-scales of social phobia did not have significant correlations with the contexts of interpersonal conversations and group discussion in L2 WTC, except that the performance avoidance in social phobia significantly correlated with group discussion in L2 WTC in a negative way. However, the sub-scales of social phobia had significant negative correlations with the contexts of meetings and public speaking in L2 WTC.

**Table 1:** Correlation analyses.

	1	2	3	4	5	6	7	8	9
1. SP-Fear-P	1								
2. SP-Fear-SI	0.910^**^	1							
3. SP-Avoidance-P	0.775^**^	0.739^**^	1						
4. SP-Avoidance-SI	0.742^**^	0.787^**^	0.923^**^	1					
5. Ideal L2 self	−0.329^**^	−0.308^**^	−0.339^**^	−0.344^**^	1				
6. L2 WTC-IC	−0.026	−0.066	−0.099	−0.085	0.211^**^	1			
7. L2 WTC-GD	−0.081	−0.106	−0.155^*^	−0.122	0.212^**^	0.755^**^	1		
8. L2 WTC-M	−0.173^*^	−0.226^**^	−0.244^**^	−0.251^**^	0.262^**^	0.703^**^	0.746^**^	1	
9. L2 WTC-PS	−0.242^**^	−0.254^**^	−0.242^**^	−0.234^**^	0.390^**^	0.613^**^	0.726^**^	0.720^**^	1

* *p* < 0.05;

** *p* < 0.01.

A series of univariate and multiple linear regression analyses were performed, and the results were exhibited in Table 2.1 and 2.2. It was revealing that the fear and avoidance subscales of social phobia had significant negative impacts on the ideal L2 self in the regression analyses (Fear subscale: *p* < 0.001; Avoidance subscale: *p* < 0.001). What stands out in Table 2.1 is that the fear and avoidance subscales of social phobia did not exert a significant influence on the contexts of interpersonal contexts (Fear subscale: *p* = 0.387 > 0.05; Avoidance subscale: *p* = 0.428 > 0.05) and group discussion (Fear subscale: *p* = 0.338 > 0.05; Avoidance subscale: *p* = 0.095 > 0.05) in L2 WTC but significantly affected the contexts of meetings (Fear subscale: *p* = 0.007 < 0.01; Avoidance subscale: *p* = 0.004 < 0.01) and public speaking (Fear subscale: *p* = 0.003 < 0.01; Avoidance subscale: *p* = 0.006 < 0.01) in L2 WTC. In other words, social phobia would undermine L2 WTC only in large meetings and public speaking, where learners face a larger audience in social situations. As denoted in Table 2.2, ideal L2 self can exert significant positive influences on all the contexts of L2 WTC (IC: *p* = 0.005 < 0.01; GD: *p* = 0.005 < 0.01; M: *p* = 0.001 < 0.01; PS: *p* < 0.001) according to the univariate regression analyses.

**Table 2.1:** Multiple regression analyses (Social phobia as independent variable).

		Social Phobia							
		**Fear**			**Avoidance**				
		**R**^**2**^	**Durbin-Watson**	**F**	***p***	**R**^**2**^	**Durbin-Watson**	**F**	***p***
Ideal L2 self		0.109	2.032	10.371	0.000^***^	0.122	2.056	11.773	0.000^***^
L2 WTC	**IC**	0.011	1.876	0.956	0.387	0.010	1.933	0.854	0.428
	**GD**	0.013	1.779	1.090	0.338	0.027	1.855	2.387	0.095
	**M**	0.057	2.098	5.131	0.007^**^	0.064	2.047	5.813	0.004^**^
	**PS**	0.065	1.946	5.931	0.003^**^	0.059	1.893	5.355	0.006^**^

** *p* < 0.01;

*** *p* < 0.001.

**Table 2.2:** Univariate regression analyses (Ideal L2 self as independent variable).

		Ideal L2 self			
		**R**^**2**^	**Durbin-Watson**	**F**	***p***
L2 WTC	**IC**	0.045	1.951	8.003	0.005^**^
	**GD**	0.045	1.860	8.046	0.005^**^
	**M**	0.068	2.111	12.560	0.001^**^
	**PS**	0.152	2.016	30.605	0.000^***^

** *p* < 0.01;

*** *p* < 0.001.

### Path analysis

**Figure 2:**
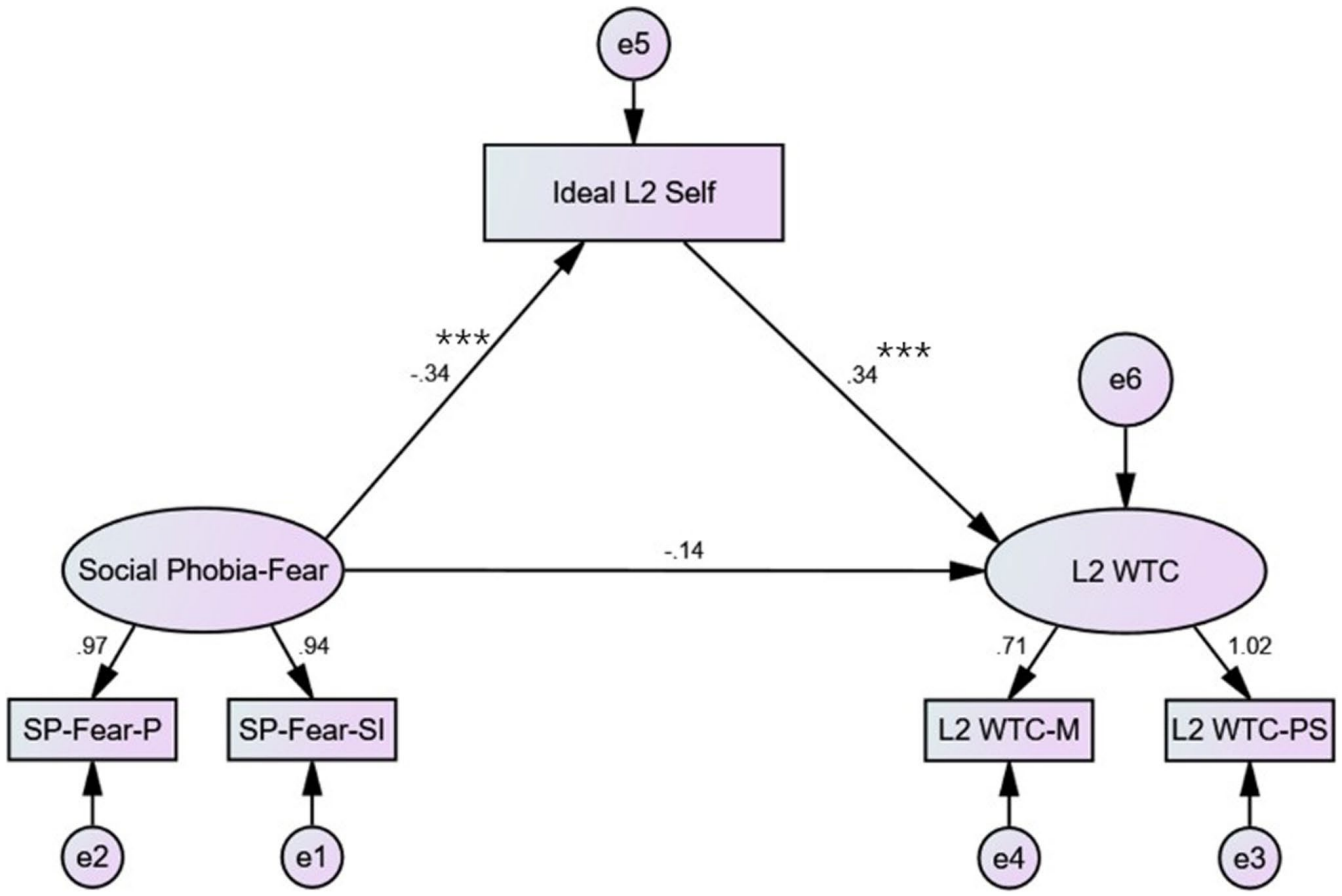
Path analysis – Social phobia-fear; ***p < 0.001.

**Figure 3:**
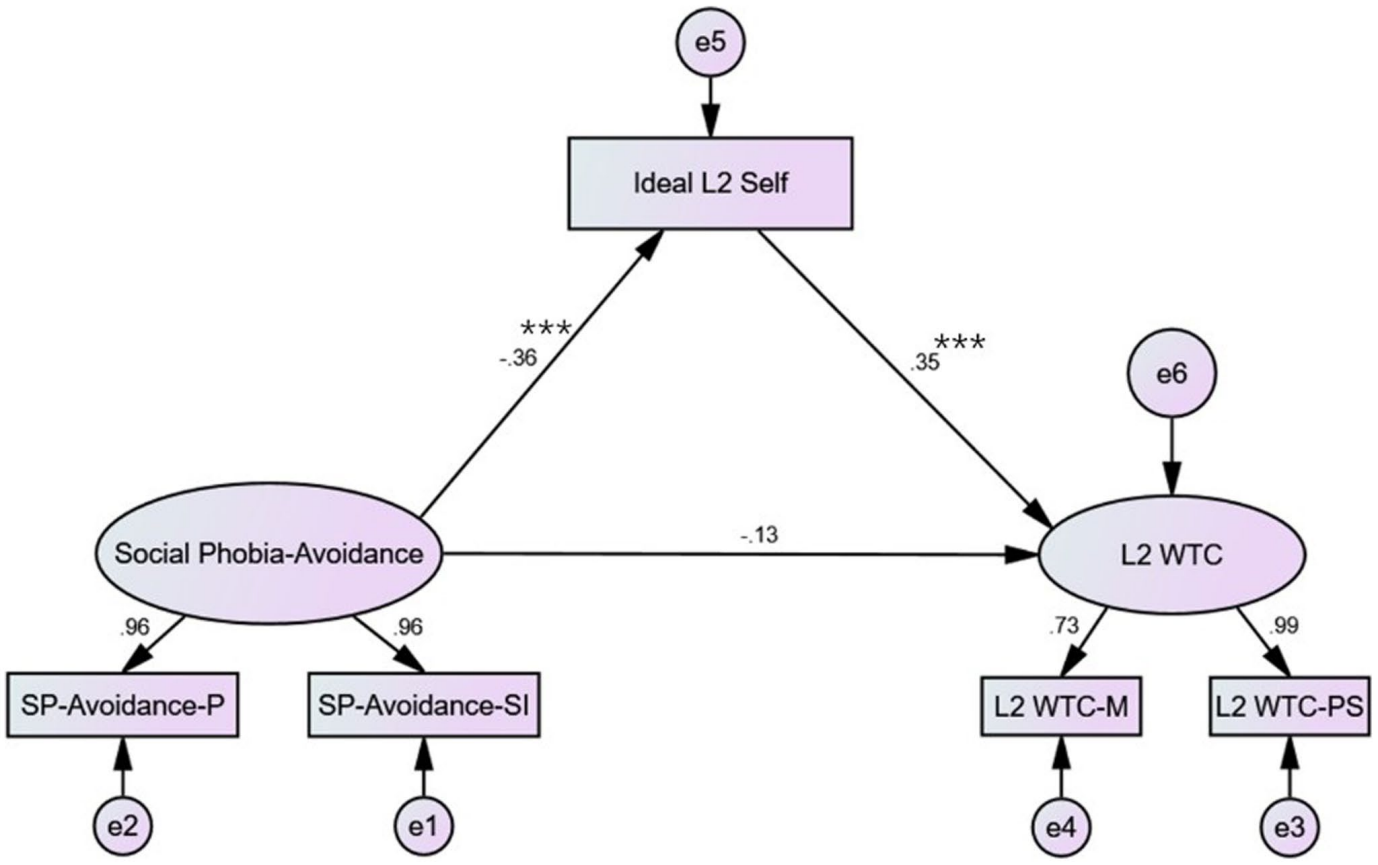
Path analysis – Social phobia-avoidance; ***p < 0.001.

The variables of the current research were further integrated into structural equation modelling analyses to delineate the path of influence by utilizing *Amos 22* (see Figures 2, 3). The data input in the SEM models were preprocessed to be standardized. Since the subscales of social phobia did not significantly affect interpersonal conversations and group discussion contexts in L2 WTC, these two contexts were excluded from the model construction.

Figures 2, 3 illustrate the influential paths of the fear subscale and the avoidance subscale of social phobia on L2 WTC via ideal L2 self, respectively. According to the path analyses, the path coefficients were significant from social phobia to ideal L2 self (*p* < 0.001) and from ideal L2 self to L2 WTC (*p* < 0.001) in both subscales. However, the path coefficients were not statistically significant from social phobia to L2 WTC directly in both subscales (*p* > 0.05). As denoted in Tables 3, 4, the indicators of data fitting were all within the reference range. Hence, the established two SEM models are optimal. The effect analyses (Tables 5, 6) reported that all the standardized total and indirect effects were significant (i.e., 0 was not involved in the 95% confidence interval). However, the standardized direct effects from social phobia to L2 WTC were not significant (i.e., 0 was involved in the 95% confidence interval). In line with findings regarding the analysing model of mediating effect from Wen and Ye (2014) the ideal L2 self acted as a complete mediating role in the impact of social phobia on L2 WTC.

**Table 3:** Data fitting in the structural equation model (social phobia-fear).

Indicators	CMIN/DF	***P***	GFI	CFI	IFI	RMR	RMSEA
Value	1.634	0.179	0.989	0.996	0.996	0.016	0.061
Reference range	≤5	>0.05	≥0.9	≥0.9	≥0.9	≤0.10	≤0.08

**Table 4:** Data fitting in the structural equation model (social phobia-avoidance).

Indicators	CMIN/DF	***P***	GFI	CFI	IFI	RMR	RMSEA
Value	1.173	0.318	0.992	0.999	0.999	0.026	0.032
Reference range	≤5	>0.05	≥0.9	≥0.9	≥0.9	≤0.10	≤0.08

**Table 5:** Effect analysis of the paths (Social phobia-fear).

Path	Standardized total effect		Standardized direct effect		Standardized indirect effect	
	95% CI		95% CI		95% CI	
	Lower limit	Upper limit	Lower limit	Upper limit	Lower limit	Upper limit
Social Phobia-Fear➛Ideal L2 self	−0.478	−0.177	−0.478	−0.177	–	–
Ideal L2 self➛L2 WTC	0.172	0.467	0.172	0.467	–	–
Social Phobia-Fear➛L2 WTC	−0.438	−0.078	−0.322	0.014	−0.191	−0.044

**Table 6:** Effect analysis of the paths (Social phobia-avoidance).

Path	Standardized total effect		Standardized direct effect		Standardized indirect effect	
	95% CI		95% CI		95% CI	
	Lower limit	Upper limit	Lower limit	Upper limit	Lower limit	Upper limit
Social Phobia-Avoidance➛Ideal L2 self	−0.495	−0.196	−0.495	−0.196	–	–
Ideal L2 self➛L2 WTC	0.170	0.473	0.170	0.473	–	–
Social Phobia-Avoidance➛L2 WTC	−0.438	−0.044	−0.334	0.053	−0.202	−0.050

## Discussion

### The effective power of social phobia on L2 WTC

The present study made an attempt to introduce social phobia, a psychopathological property to second language research and explored the effective power of social phobia on L2 WTC. The results of this interdisciplinary investigation revealed that social phobia, including the fear and avoidance subdivisions, had a significant negative impact on L2 WTC. However, this critical impact was only prominent in the social situational contexts of large meeting and public speaking. By way of explanation, social phobia could be a strong negative predictor of L2 WTC only when individuals face a larger audience.

This result further confirmed the adverse influence of social phobia on academic performance (Baptista et al., 2012; Leigh et al., 2021; Vilaplana-Pérez et al., 2021; Archbell and Coplan, 2022) and on L2 acquisition and learning (King, 2013a,b). Meanwhile, the current research provided much empirical evidence and specifically elucidated how and to what extent social phobia could affect L2 WTC. It was quite interesting to detect that social phobia would not be a salient attribute of L2 WTC when the audience size is small (i.e., in interpersonal or small group scenarios). However, it would significantly cloud the outlook of L2 WTC when there is a mass audience (i.e., in large meetings and public speaking settings).

A possible explanation for this might be that the sufferers’ cognitive resources are overloaded when processing the potential feedbacks from a larger audience. Cognitive load theory posited that the cognitive-processing capacity is rather limited, and this is in line with the limitation of working memory (Sweller, 1988). When social phobics use an L2 to perform or interact in social encounters, their intrinsic cognitive load (i.e., demanded by the basic structure of information, Sweller et al., 2011) and extraneous cognitive load (i.e., demanded by the manner how the information is presented, Sweller et al., 2011) would considerably increase. Although people suffering from social phobia usually hold negative self-beliefs and misconceptions of their abilities, they set extremely high standards, hoping to achieve perfection in L2 expression and behavioural propriety in social scenarios. Therefore, on the one hand, they would particularly concentrate on the correctness and appropriateness of their L2 expressions and the potential direct and indirect language-related feedbacks from the audience. On the other hand, they feel obliged to digest the potential scrutiny and negative evaluations in terms of their social behaviours from the audience as well. If they unintentionally made a mistake in L2 delivery or improperly behaved, their negative self-appraisal would be immediately triggered, accompanied by a series of somatic symptoms and rumination. All these efforts would occupy and squeeze the limited cognitive-processing capacity (Moriya and Sugiura, 2013). When social phobics are engaged in interpersonal conversations or small group discussion, it is possible that their cognitive resources are still adequate, since the potential feedbacks would not be prolific. However, when sufferers of social phobia were immersed in larger social contacts, their cognitive-processing capacity would soon be exhausted. Their fear and avoidance of social performances and interactions could be instantly aroused. Thus, their willingness to communicate, especially using an L2, is strongly affected in a negative way.

### Ideal L2 self as a mediating factor

Apart from the critical overall impact of social phobia on L2 WTC, this research also innovatively examined the influential trajectory from social phobia to L2 WTC, via ideal L2 self. The result highlighted the complete mediation effect of ideal L2 self and demonstrated that ideal L2 self is an indispensable variable to consider in the impact.

In the current study, it was pinpointed that social phobia could substantially impair L2 WTC, by first exerting an undermining effect on ideal L2 self. In other words, though social phobia could significantly influence the level of L2 WTC in social settings of large meetings and public speaking, it could only exert its impact indirectly via ideal L2 self. This finding is meaningful, since it informed us that social phobia and L2 WTC did not enjoy a simple direct linear relationship. Social phobics tend to avoid potential negative outcomes and are characterized by behavioural inhibition (Rosenbaum et al., 1991; Neal et al., 2002). People who suffered from social phobia usually withdraw from social communications because of their constant and excessive fear regarding the potential negative evaluations and embarrassing observations (Hudson and Rapee, 2000). This then would elicit behavioural inhibition and demotivation in social situations [American Psychiatric Association (APA), 2000], especially when an L2 is possibly used. This nature of withdrawal would weaken behavioural intention and distort the self-images in L2 learning (Rapee and Heimberg, 1997; Izgiç et al., 2004). Hence, the level of ideal L2 self was decreased accordingly under the negative influence of social phobia. The finding from the current study was also in accordance with past literature on the positive relationship between ideal L2 self and L2 WTC (Munezane, 2013; Öz, 2016; Lee and Lee, 2020; Shen et al., 2020; Lan et al., 2021; Zhang et al., 2022). The adverse impact from social phobia was further transmitted to L2 WTC, based on the logical reasoning between ideal L2 self and L2 WTC. The motivation for communication in an L2 context has thus been weaken and diminished.

## Conclusion

The current study conducted an interdisciplinary investigation and obtained convincing empirical evidence. It has been one of the initial attempts to thoroughly examine the impact of domain-general psychopathological properties in second language acquisition and learning. The findings provided a deeper insight into the intriguing negative relationship between social phobia and L2 WTC and further extends our knowledge of the influential trajectory. Prior to this exploration, little was known about the potential effect of social phobia on L2 learning, thus the evidence attained proved to be eminently valuable. Meanwhile, the present research innovatively considered ideal L2 self in the impact and elucidated that the pathway of impact from social phobia to L2 WTC was not direct and linear, since ideal L2 self acted a mediating role. The in-depth methodology of data analysis was another key strength of the present research. A comprehensive picture of the data was exhibited by conducting a series of correlation, regression and structural equation modelling analyses.

This research also proffered an illuminating account on the pedagogical implications for L2 acquisition and learning. Firstly, L2 language teachers and practitioners should specifically pay attention to the students who has the inclination of social phobia and actively provide relevant support for these students. Psychotherapists could be invited to join the language practitioners to guide the students through the distress of social phobia, by imparting coping skills in social situations and supply Cognitive behavioural Therapy (CBT) or medication when appropriate. Secondly, when students who suffer from social phobia are involved in L2 social communications, teachers and practitioners should allow them to start from small-scale L2 scenarios and gradually increase their audience size. In this process, teachers should be fully aware of their possible fear and avoidance and offer directions in terms of how to minimize their unnecessary concerns about others’ negative evaluations. This approach would reduce their intrinsic and extraneous cognitive load and enhance their L2 WTC in larger-scale social encounters. Thirdly, to eventually advance sufferers’ L2 WTC, teachers could try to help them establish vivid and clear L2 self-images and motivate them to raise the level of ideal L2 self. This would be surely helpful towards improving their L2 WTC substantially.

Notwithstanding the key empirical findings in the current study, a number of limitations need to be noted. Firstly, this research mainly probed into how social phobia, as an independent variable, would influence L2 WTC. The possibility of social phobia related comorbidity was not included in the research design. Secondly, the proficiency level of L2 learners was not included as a grouping variable, so a fine-grained picture of the impact of social phobia on L2 WTC remains blurred. Thirdly, when examining L2 WTC, only the categorization of different social scenarios was considered. Later studies could endeavour to gain further insights into the effect of social phobia on L2 WTC according to the categorization of the intimacy between the sufferer and the audience.

Considerably more work will need to be done to assess the longitudinal and dynamic changes of the impact of social phobia on L2 WTC. Additionally, it is worth exploring the impact of social phobia on other aspects of L2 acquisition and learning, to enrich the interdisciplinary investigation between psychopathology and L2 learning. Furthermore, qualitative examinations would be valuable and beneficial to contribute to and verify the existing evidence.

Generally, L2 learners with lower level of social phobia would have a more positive outlook concerning ideal L2 self-images and the behavioural intention to perform and interact in social settings. This would further advance learners’ level of L2 WTC and contribute to more possible progress and development in L2 acquisition and learning.

## Data availability statement

The original contributions presented in the study are included in the article/supplementary material; further inquiries can be directed to the corresponding author.

## Ethics statement

The studies involving human participants were reviewed and approved by College of Foreign Languages, Nankai University. The patients/participants provided their written informed consent to participate in this study.

## Author contributions

CZ contributed to the conceptualization of the study, literature review, data collection and analysis, and writing the first draft. WZ contributed to the conceptualization of the study, reviewing and editing. All authors contributed to the article and approved the submitted version.

## Funding

This work was supported by Project of Discipline Innovation and Advancement (PODIA)—Foreign Language Education Studies at Beijing Foreign Studies University [Grant number: 2020SYLZDXM011], Beijing; and the Tianjin Philosophy and Social Science Planning Research (Youth Project), entitled “The impact of college students’ psychological wellbeing on foreign language learning” [Grant number: TJJXQN19-001].

## Conflict of interest

The authors declare that the research was conducted in the absence of any commercial or financial relationships that could be construed as a potential conflict of interest.

## Publisher’s note

All claims expressed in this article are solely those of the authors and do not necessarily represent those of their affiliated organizations, or those of the publisher, the editors and the reviewers. Any product that may be evaluated in this article, or claim that may be made by its manufacturer, is not guaranteed or endorsed by the publisher.
